# Into the understanding the multicellular lifestyle of *Proteus mirabilis* on solid surfaces

**DOI:** 10.3389/fcimb.2022.864305

**Published:** 2022-09-02

**Authors:** Dawid Gmiter, Wieslaw Kaca

**Affiliations:** Deparment of Microbiology, Institute of Biology, Faculty of Natural Sciences, Jan Kochanowski University in Kielce, Kielce, Poland

**Keywords:** *Proteus mirabilis*, swarming motility, biofiolm, urinary tract infections (UTIs), bacterial interactions

## Abstract

Indwelling urinary catheterization can lead to the development of catheter-associated urinary tract infections (CAUTIs), an important type of nosocomial infection, as well as other medical issues among institutionalized adults. Recently, *Proteus mirabilis* was highlighted as the important cause of CAUTIs. The pathogenicity of *P. mirabilis* is dependent on two multicellular types of surface colonization: the adherence and swarming motility. Adhesion, mostly mediated by fimbrial and nonfimbrial adhesins, is important for the initiation of biofilm formation. Moreover, the production of urease frequently results in biofilm crystallization, which leads to the blockage of catheters. The heterologous polymeric matrix of the biofilm offers protection against antibiotics and the host immune system. *P. mirabilis* displays remarkable motility abilities. After contact with solid surfaces, hyper-flagellated cells are able to rapidly migrate. The importance of swarming motility in CAUTIs development remains controversial; however, it was indicated that swarming cells were able to co-express other virulence factors. Furthermore, flagella are strong immunomodulating proteins. On the other hand, both biofilm formation and swarming motility implicates multiple inter- and intraspecies interactions, which might contribute to the pathogenicity.

## Introduction

Surface colonization by bacteria allows multicellularity. The properties of biofilm formation and swarming motility both require direct cooperation and communication between individual cells, which in turns leads to an intracellular change compared with a self-inhabiting cell. These two methods of bacterial surface colonization show distinct mechanisms of action and regulation (Verstraeten et al., 2008); however, the bacterium *Proteus mirabilis* is known to utilize both processes during pathogenesis and bacterial interactions, mostly during catheter-associated urinary tract infections (CAUTIs) (Rózalski et al., 1997; Pearson et al., 2008).

*P. mirabilis*, discovered by the German scientist Gustav Hauseris, is a Gram-negative rod-shaped bacterium originally classified in the Enterobacteriaceae family. However, in 2016, the genus Proteus was reclassified into the new family Morganellaceae (Armbruster and Mobley, 2012; Armbruster et al., 2018). *P. mirabilis* is a facultative anaerobe that can utilize citrate, does not decompose maltose and lactose, and does not produce indole (Rózalski et al., 1997; Umar et al., 2016). The bacterium is abundant in the natural environment, reflecting its ability to metabolize organic substances, and is also part of the normal human intestinal flora (Hayder et al., 2020; Yuan et al., 2021). *P. mirabilis* is an opportunistic, conditional pathogen, that causes infections in immunocompromised individuals. It is also a common cause of nosocomial infections, including infections of the urinary tract, wounds, and blood. Although, bloodstream infection due to *P. mirabilis* strains is a relatively uncommon clinical entity (Endimiani et al., 2005).

*P. mirabilis* is significantly associated with urinary tract infections (UTIs), which are common bacterial infections affecting approximately 150 million people each year worldwide (Flores-Mireles et al., 2015; Yuan et al., 2021). UTIs are classified into uncomplicated and complicated infections. The latter applies to patients with anatomical or functional abnormalities of the urinary tract and patients with long-term catheters who may develop catheter-associated urinary tract infections. CAUTIs account for up to 80% of all in-hospital UTIs (Armbruster and Mobley, 2012; Flores-Mireles et al., 2015). They present a serious problem in nursing homes for patients > 65 years of age. Research shows that older people in institutional care are more likely to develop UTIs, including CAUTIs (Rowe and Juthanu-Mehta, 2013). However, it should be noted that most cases of catheter-associated bacteriuria did not progress to symptomatic UTIs (Jacobsen et al., 2008).

*P. mirabilis* is a particularly important etiological factor in patients with complicated UTIs (Chen et al., 2012; Schaffer and Pearson, 2015) and plays an significant role in CAUTIs, contributing to 5-44% of this type of infection (Coker et al., 2000; Schaffer and Pearson, 2015; Flores-Mireles et al., 2019). This bacterium is also commonly isolated in CAUTIs cases among nursing home residents (Armbruster et al., 2017). The opportunistic nature of *P. mirabilis* bacteria results from numerous pathogenic factors that ensure the virulence of cells at every stage of infection. Factors such as swarming motility, enzyme activity (urease, protease, and haemolysin), fimbriae and biofilm formation, as well as lipopolysaccharide synthesis, all play a role (Rózalski et al., 1997; Armbruster and Mobley, 2012; Armbruster et al., 2018).

**Figure 1:**
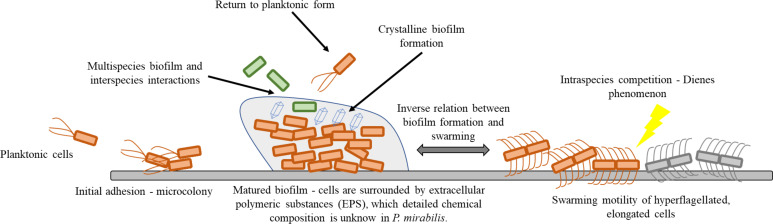
Schematic presentation of *Proteus mirabilis* lifestyle on solid surfaces.

Within this review, we focus on the adhesion, biofilm formation, and swarming motility of *P. mirabilis*. We briefly describe basics mechanisms of mentioned properties and summarize the newest discoveries related to these processes. The review also raises the issues of inter- and intra-bacterial interactions, being a consequence of the multicellular nature of the discussed forms of cell existence. The focus of this review is illustrated in **Figure 1**.

## Adhesion and biofilm formation

UTIs caused by *P. mirabilis* are most often ascending, i.e., bacterial cells must pass (e.g., from the gastrointestinal tract) to other regions of the urinary system (Armbruster and Mobley, 2012). The key to initiating infection is firstly adhesion of cells to biotic and/or abiotic surfaces (which in the case of *P. mirabilis*, often involves a catheter). *P. mirabilis* cells also adhere to the body fluids-derived protein coated on catheter surfaces (Wasfi et al., 2020). Adhesion occurs through specialized fimbrial and non-fimbrial adhesins. Regardless of the mechanism of bonding to the surface, interactions usually originate from the same fundamental physicochemical forces, and cells bind to the substrate via nonspecific, reversible interactions such as gravitational, electrostatic, and hydriophobic van der Waals forces (Berne et al., 2015).

Study shown high adherence to the catheter of *P. mirabilis* in comparison to other Gram-negative bacteria. Fimbria play a pivotal role in *P. mirabilis* cells adhesion on the surfaces. Genome analysis of the *P. mirabilis* HI4320 strain revealed the presence of 17 genes clusters encoding fimbrial proteins, a larger number than among other pathogenic bacteria (Pearson et al., 2008; Kuan et al. 2014; Wasfi et al., 2020). Greek classification classify fimbriae into nine groups (α, β, γ1, γ2, γ3, γ4, κ, π and σ). In *P. mirabilis* all fimbriae, except fimbriae 14, were classified into three clades (γ1, γ2, π). The prototypical member of this clade could be found in the uropathogenic E. coli. Additionally, study revealed high level of conservatism of fimbriae related genes among *P. mirabilis* isolates (Kuan et al., 2014).

To date, however, most of the studies focused on five types of fimbriae expressed by the *P. mirabilis* (Rózalski et al., 1997; Rocha et al., 2007; Pearson et al., 2008; Kuan et al., 2014). These fimbriae play a different roles in the adhesion to host cells and/or the surface of catheters, and also in the process of biofilm formation (Rocha et al., 2007; Scavone et al., 2016). Their importance is presented in **Table 1**. The literature also highlights the potential importance of fimbria 14, which was discovered during genome analysis of *P. mirabilis* HI4320 strain. The exact role of this fimbria is unknown, but its overexpression was observed during infection and at pH 8.0 (an alkaline pH is generated by the enzyme urease) (Kuan et al., 2014; Schaffer and Pearson, 2015). Sequence analysis of the incomplete genome of *P. mirabilis* Pr2921 strain indicated the presence of genes encoding fimbria-related proteins that are not present in strain HI4320, although they are present in other *P. mirabilis* strains (Giorello et al., 2016).

**Table 1:** Characteristics of most studied *Proteus mirabilis* fimbriae.

Fimbriae	Biological role
Mannose-resistant/*Proteus*-like fimbriae (MR/P)	The mutant strains showed lower adhesion rates than the strain *P. mirabilis* wild-type and were significantly less effective in inducing genotoxic and cytotoxic effects compared to the wild-type. The mutants also showed a lower colonization efficiency of the lower and upper urinary tract, although their virulence was not reduced.
*P. mirabilis* fimbriae (PMF)	PMFs play a role in bladder colonization, while the issue of kidney colonization by strains lacking PMF is debatable.
Uroepithelial cell adhesins (UCA)/nonagglutinating fimbriae (NAF)	They play a role in adhesion to urinary tract epithelial cells *in vitro*. UCA fimbriae play an important role in the colonization of the urinary tract.
Ambient-temperature fimbriae (ATF)	ATF fimbriae play a role in *P. mirabilis* survival in the external environment due to the optimal expression temperature.
*P. mirabilis* P-like pili (PMP)	PMP fimbriae have been suggested to play a role in the adhesion of *P. mirabilis* in the bladder and kidneys

Based on: Rózalski et al. (1997); Rocha et al. (2007); Kuan et al. (2014); Schaffer and Pearson (2015); Scavone et al. (2016).

The initial adhesion of *P. mirabilis* cells to biotic and abiotic surfaces leads to the formation of a biofilm by the bacteria. Biofilm is a complex structure in which bacterial cells are surrounded by biopolymers, extracellular polymeric substances - EPS. Biofilm is an excellent form of protection against external physical, chemical, and biological factors, including antimicrobial compounds. For this reason, biofilm presents a significant medical problem. In the case of *P. mirabilis*, biofilm is most often formed on the surfaces of catheters. As with other biofilm-forming bacteria, biofilm formation by *P. mirabilis* is associated with greater antimicrobial resistance (Aiassa et al., 2006). EPS consists mainly of polysaccharides, proteins, extracellular DNA, glycoproteins, lipids, lipoteichoic acids, and lipopolysaccharides (LPS). Water, which plays a protective role, also constitutes a considerable proportion of the biofilm matrix (approximately 97%) before drying. The average thickness of a biopolymer layer is 0.2–1.0 mm (Boudarel et al., 2018; Czyzewska-Dors et al., 2018).

The composition of the EPS defines the biofilm properties (Boudarel et al., 2018). However, the components of *P. mirabilis* EPS have not been determined in detail. In a study by Zhang et al. (2009), EPS produced by *P. mirabilis* TJ-1 strain was used for the removal of a hazardous dye, basic blue 54 (BB54). They reported that the *P. mirabilis* TJ-1 EPS consisted of 30.9% protein and 63.1% polysaccharide. FTIR spectra revealed the presence of amino, hydroxyl, and carboxyl groups. Intriguingly, the study of *P. mirabilis* 9B-m (O11a) LPS isolated from planktonic, sessile, and biofilm-forming cells, revealed a reduction in the O-polysaccharide component of LPS in the biofilm-forming cells compared with the other cell types. In sessile cells, the core oligosaccharide of LPS possessed additional acetylated hexosamines. In contrast to the polysaccharide region of LPS, the lipid A structure was conserved among the cells types (Zabłotni et al., 2018). Some details about the structure of *P. mirabilis* biofilm were presented by Jones et al. (2007), who compared the biofilm formed in artificial urine and Luria–Bertani broth using CSLM and 3D imaging. It was demonstrated that, depending on used media, two markedly different biofilm structures were formed. Biofilms grown in Luria-Bertani broth formed mushroom structures at 24 h and contained nutrient channels, meanwhile, biofilm structure in artificial urine medium was observed to be a flat layer, almost devoid of nutrient channels.

A unique characteristic of *P. mirabilis* is the formation of a so-called crystalline biofilm, which can lead to incrustation, i.e., the blocking of catheters by the formation of crystals (Yuan et al., 2021). This process involves the crystallization of struvite and apatite carbonate in the urine at an elevated pH, which is generated by the urea decomposition reaction mediated by the enzyme urease. Blocking the proper flow of urine through a catheter and the resulting urine retention is painful for patients and can lead to serious complications, including pyelonephritis and even septic shock (Wilks et al., 2015). The importance of urease-producing bacteria, such as *P. mirabilis*, and related factors in the biomineralization of struvite was previously reviewed by (Pelling et al., 2019) and (McLean and Brown, 2020). It is worth mentioned that Wilks et al. (2015) revealed four stages of crystalline biofilm formation by *P. mirabilis* using a non-contact, non-destructive real-time imaging method. In summary, these are: (1) an initial colonization by bacteria surrounded by large amounts of sugar-based carbohydrates, (2) formation of a sheet-like microcrystalline material (3) accumulation of diffuse crystalline material and (4) formation of biofilm containing defined crystals and highly motile *P. mirabilis* cells.

Despite above mentioned, still little is known about the molecular mechanisms involved in *P. mirabilis* biofilm formation. Some important factors (and the methods of biofilm development control) were summarized in 2011 by Jacobsen and Shirtliff, 2011 and recently by Wasfi et al. (2020) and Yuan et al. (2021). However, these reports do not cover all discoveries in the field.

For example, recent works revealed the role of two-component signal transduction systems in the process. Firstly, Howery et al. (2016) showed that a rcsB mutant strain was impaired in biofilm formation. The rcsB and rcsC are elements of the regulator of colonic acid capsule synthesis (Rcs) phosphorelay, complex signal transduction system present in many members of the Enterobacteriaceae. In *P. mirabilis*, Rcs system is considered most often as a regulator of swarming motility, as it controls, among others, differentiation into the swarming cells through the repression of the flhDC operon and activation of the minCDE cell division inhibition system (Pearson et al., 2008; Howery et al., 2016). As indicated using RNA-Seq, the RcsB regulator influence the expression of mrpA, pmfA, and ucaA genes (involved in the formation of fimbriae) and outer membrane protein A (ompA) gene (shown to bind abiotic surfaces). Nevertheless, the exact mechanism leading to the biofilm deficient phenotype in rcsB mutant remains unknow. This data, however, in combination with observation that rcsD (also known as rsbA in *P. mirabilis*) mutant is deficient in biofilm production (Liaw et al., 2004), support the importance of Rcs phosphorelay in *P. mirabilis* biofilm formation.

Another example showing the relevance of two-component systems was presented by Chen et al. (2020). The study revealed the role of the CpxR-regulated zapD gene in biofilm formation. The CpxAR system is one of the most widespread two-component signal transduction systems in Gram-negative bacteria. Meanwhile zapD gene encodes an outer membrane protein of the putative type 1 secretion system ZapBCD. As indicated, the loss of cpxR or zapD resulted in a reduction in biofilm formation ability, and mutants showed significantly lower protease activity, adhesion, auto-aggregation, and exopolysaccharide and extracellular DNA levels compared with the wild-type. Intriguingly, it was shown that CpxR-dependent biofilm formation was regulated by the presence of copper (Chen et al., 2020).

The results above shed light on the role of mentioned two-component systems. Despite being investigated as a regulators of swarming motility, little is known about its role in biofilm formation and further studies are still required. Moreover, 16 different two-component systems were identified in the genome of *P. mirabilis* strain HI4320, which were not tested to this day (Pearson et al., 2008).

Further, Debnath et al. (2018) presented new inside into the intracellular regulation of fimbriae in *P. mirabilis*. They reported the set of virulence related genes directly regulated by MrpJ using Chromatin immunoprecipitation followed by high-throughput sequencing (ChIP-seq). This work details an earlier observation from the microarray-based approach which identified genes regulated by MrpJ in an indirect and direct way. The mutational approach, involving construction of both single and multiple genes mutants, demonstrated that novel MrpJ fimbrial targets contribute to *P. mirabilis* pathogenesis. Obtained result, therefore, contribute to the better understanding of factors involved in adherence and biofilm formation in *P. mirabilis* virulence. In connection with above mentioned, Jiang et al. (2020) revealed the role of zinc-binding protein MrpH, a new class of metal-binding adhesin, in mediating biofilm formation by *P. mirabilis*. The mrpH gene is a part of the MR/P fimbriae operon. The study shown that zinc plays a key role in transition from planktonic into adhered cells.

Furthermore, zinc and copper are not the only metal ions having role in the *P. mirabilis* biofilm formation. The results of Iribarnegaray et al. (2021) revealed the relevance of iron metabolism in biofilm and infection development. The results obtained confirm the relevant role of ferritin and a TonB-associated porin protein in *P. mirabilis* pathogenicity.

Recent studies have also focused on investigating the mechanisms that contribute to *P. mirabilis* biofilm maturation and functionality. Maszewska et al. (2021), using a proteomics approach, identified proteins potentially participating in antibiotic and phage resistance of *P. mirabilis* cells residing in a mature biofilm. Meanwhile White et al. (2021) revealed that catalase activity, driven by the katA gene, is crucial for biofilm formation and regulates the EPS composition (as katA deletion reduced the carbohydrate content). This modification resulted in the decreased antibiotic resistance and virulence of katA mutants.

Taken together, these findings extend our understanding of biofilm formation by *P. mirabilis*, which may lead to the development of novel antibacterial strategies. However, many aspects of biofilm formation, particularly the role of all 17 fimbriae in adhesion, gene regulation and the EPS composition, remain to be explored. Better understanding of regulatory mechanisms involved in *P. mirabilis* biofilm formation could be obtained through the analysis of transcriptome changes during the adhesion, as it was done by Jones et al. (2022), who tested the Pseudomonas aeruginosa response to surfaces using RNA-Seq.

## Swarming motility

Swarming motility is a method of migration of flagellated bacterial cells over solid and semi-solid surfaces (Verstraeten et al., 2008). The ability to swarm is one of the characteristics of *P. mirabilis* and was first observed and described in 1885 by Gustav Hauser (Pearson et al., 2008). The swarming motility of *P. mirabilis* can be easily observed under laboratory conditions using media enriched with microbial agar at concentrations of 1.5%–2%. Upon inoculation of *P. mirabilis* cells, which are in the form of swimmer cells in liquid culture, they transform into cells capable of migration. Such migratory cells have a distinct morphology, taking the form of long cells with an increased number of flagella and nucleoids not separated by a septum. Cell migration is interspersed with periods of so-called consolidation. During this time, individual migrating cells that have “moved away” from the point of initiation of creeping growth, break up. After reaching the appropriate density within the area of consolidation, re-transition to a cellular phenotype capable of swarming motility occurs, and cells undergo migration. Both stages are repeated cyclically during incubation, so swarming motility is observed in the Petri dish in the form of alternating concentric circles. This pattern is referred to as the “bull’s eye pattern” (Rózalski et al., 1997; Jones et al., 2004; Pearson et al., 2008; Armbruster et al., 2018).

The role of swarming motility in the pathogenesis of *P. mirabilis*, however, remains to be fully elucidated. It is assumed that swarming enables colonization of the upper urinary tract in individuals undergoing catheterization, as *P. mirabilis* cells are able to grow and spread across the surface of latex and silicone catheters. Additionally, in swarmer cells, overexpression of ureolytic, proteolytic, and haemolytic activities is observed. Therefore, it is conceivable that the co-expression of enzymes essential for the development of infection in migrating cells allows *P. mirabilis* to establish a CAUTIs. Moreover, transposon mutants that were mobile but did not show swarming ability resulted in lower rates of mortality and renal abscesses in the mouse UTIs model. Another study indicated that swarmer cells were rarely detected in the urinary tract of CBA/J mice infected (by bladder catheterization) with *P. mirabilis* cells labelled with green fluorescence protein when tested two or four days after infection (Schaffer and Pearson, 2015). It has also been shown that neither swarming nor swimming motility reduced *P. mirabilis* adhesion, and strains deficient in swarming and swimming were also capable of forming crystalline biofilms and blocking catheters more rapidly than the wild-type strain (Jones et al., 2005). It is noteworthy that the synthesis of flagella, which are responsible for the migration process, is increased in the case of swarming cells, and flagella are effective in stimulating a humoral response by the host’s immune system. Their synthesis is regulated by the flhDC genes. Increasing the expression of these genes leads to an increased number of flagella on bacterial cells and the transformation of short-form cells into elongated forms (Morgenstein et al., 2010). The transformation into “swarmer cells” and the process of flagella synthesis are regulated by numerous extracellular and intracellular factors that were reviewed previously (Rather, 2005; Morgenstein et al., 2010). Swarming motility requires drastic changes in *P. mirabilis* cell morphology, which are coupled with changes in gene expression patterns. A comparison of the transcriptomes of cells at consolidation phase with those capable of swarming motility revealed a number of differentially expressed genes between the two cell types (Pearson et al., 2010).

Despite research being conducted for a decades on the mechanisms of swarming motility in *P. mirabilis*, recent studies have revealed new aspects of the process. Little et al. (2018) demonstrated the role of the rffG gene in regulating the swarming ability of *P. mirabilis* cells. This gene encodes a protein homologous to the dTDP-glucose 4,6-dehydratase protein of Escherichia coli that plays a role in the biosynthesis of enterobacterial common antigen. Strains lacking the rffG gene, as a result of stress caused by the incorrect formation of the cellular envelope, lose the ability to form cells capable of swarming motility. Another study by Little et al. (2019) investigated the influence of the surface on the swarming motility of *P. mirabilis*. It was shown that under favourable conditions (appropriate agar concentrations), cell elongation and mobility were coupled with population migration. It has also been shown that undisturbed LPS synthesis was not a key factor in *P. mirabilis* cell swarming motility, but rather a factor that allows for an increased agar concentration range in the medium, which in turn, allowed for the coupling of cell elongation and mobility with population migration.

Another area of interest is the natural diversity of *P. mirabilis* in terms of swarming ability. (Fusco et al., 2017; 2018) compared selected virulence factors of two strains of *P. mirabilis* that were extremely different in their ability to swarm. It was shown that the strain with limited ability to migrate was characterized by lower expression of proteolytic activity, which caused it to induce a different mechanism of apoptosis in human prostatic adenocarcinoma PC-3 compared with the strain showing the degree of migration ability characteristic of *P. mirabilis*. Results revealed that in the presence of low swarming strain the intrinsic pathway factors (BCL-2, BAX, and Caspase-9) related genes were upregulated. Meanwhile, strains with hight swarming ability modulates positively the expression of the genes that encode the apoptotic factors of the extrinsic pathway. Additionally, study found that the genes belonging to the execution pathway are upregulated by both strains, however, modulation was much more apparent in the second one (Fusco et al., 2018). This result demonstrated that understanding the specific relationships between virulence factors allowed the possibility of using personalized therapy to target different virulence factors. This idea is supported by the work of Peng et al. (2020) who performed a comparative analysis of selected virulence factors in two *P. mirabilis* strains characterized by limited swarming ability. RNA-Seq analysis of the transcriptome of the strains, performed in comparison with the reference strain HI4320, revealed lower expression of genes related to the synthesis of flagella and fimbriae, the transport of dicarboxylates, and the metabolism of cystathionine and anthranilate in the test strains compared with control strain HI4320. In contrast, genes related to iron transport, molybdenum metabolism, and metalloprotease were upregulated. Also our recent research focused on the diversity of two strains of *P. mirabilis* regarding their territorial capacity, both in terms of the kinship identification process and the ability to occupy the surface (Gmiter et al., 2019). The mechanism responsible for the reported divergence remains unexplained, and might not be limited to already known factors. The discovery of new regulators of swarming motility might be useful in the design of new therapeutics.

As mentioned, Rcs two-component system plays an important role in regulation of swarming motility, being negative regulator of flhDC gene expression. Further, Şimşek et al. (2021) investigated the role of the Rcs regulatory system on the surface expansion of *P. mirabilis* bacterial communities in the context of partial migration using quantitative microscopic imaging supported by direct cell labelling with the transcriptional fusion of the flhDC and green fluorescent protein (gfp) genes. Their findings illustrate the importance of Rcs in regulation of the dynamics between motile and non-motile *P. mirabilis* cells during surface colonization.

Apart from Rcs regulon, study of Wang et al. (2008) indicated the importance of RppA from the RppBA two-component regulatory system in the regulation of polymyxin B susceptibility, swarming, and virulence factor expression in *P. mirabilis* strain N2. It was observed that the rppA knockout mutant exhibited greater swarming motility and cytotoxic activity and expressed higher levels of flagellin and hemolysin comparing to the wild type. This suggests that RppA negatively regulates swarming, hemolysin expression, and cytotoxic activity.

Meanwhile, the most recent study revealed that the QseEF two-component system-GlmY Small RNA (sRNA) regulatory pathway controls the *P. mirabilis* swarming. This is the first report in which a pathway mediated by a two-component system through an sRNA was disclosed to be involved in swarming migration (Lin et al., 2022). This observation is intrigue given the fact that in silico study reported the detection of 14 sRNA candidates, conserved in the orthologous regions of *P. mirabilis*, which biological role remains unexplained (KishanRaj et al., 2018).

## Intra- and interspecies interactions

Swarming motility is most often considered in the context of its role in the pathogenicity of *P. mirabilis*. However, it is also related to an ecologically interesting feature of this species, known as the Dienes phenomenon. This refers to the ability of two strains of *P. mirabilis* to form the Dienes line (also known as the demarcation line) (Gibbs and Greenberg, 2011; Gibbs et al., 2011; Alteri et al., 2017). The Dienes line is a macroscopically observable line formed at the point of colony contact of two strains capable of migration on solid surfaces. The strains that form the Dienes line are defined as unrelated. Whereas, when colonies merge freely with each other, we refer to the kinship of the strains (Gibbs et al., 2011; Alteri and Mobley, 2016; Drzewiecka, 2016). The test determining the relationship between strains is called the Dienes test, and this has become a quick and useful method of strains differentiation under laboratory conditions (Pfaller et al., 2000).

In the early research relating to this phenomenon, the role of bacteriocins produced by cells was suggested. However, studies have shown that the ability to produce bacteriocins and the sensitivity to these compounds do not fully explain all observed cases of Dienes line formation (Budding et al., 2009). Information on the mechanisms involved in the Dienes phenomenon has only been published since the start of the 21st century. In 2009, Budding et al. demonstrated that direct contact of the cells of unrelated *P. mirabilis* strains is necessary for the formation of the Dienes line. In addition, it was observed that in the space at the Dienes line, circular cells form. Interestingly, in a pair of interacting unrelated strains, circular cells were always observed indicating that cells of one of the strains were transforming into this form and suggesting the existence of competition between the cells of the two strains. Budding et al. (2009) also noted that competition between strains occurs only under conditions that allow swarming, this effect is not observed under liquid culture conditions or during co-formation of biofilm by unrelated strains (Budding et al., 2009). Research by (Gibbs et al., 2008) revealed that the recognition of kinship between strains results from the production of specialized proteins encoded by the idsABCDEF (ids, identification of self) gene cluster. As part of their research, Gibbs et al. showed that interaction of the proteins encoded by the idsD and idsE genes was responsible for the recognition of kinship and the activation of unknown signalling pathways that lead to the formation of the Dienes line (Cardarelli et al., 2015; Saak and Gibbs, 2016). These proteins possess domains that allow for the formation of a protein complex that is the carrier of information about the relationship of strains. However, when a protein complex cannot be formed as a result of protein sequence differences, the Dienes line is formed. It was also observed that the idsA and idsB genes encode the proteins Hcp and VgrG, respectively, which are components of the type VI secretion system (TVISS) (Wenren et al., 2013; Saak and Gibbs, 2016). The structure and mode of action of TVISS were discussed in our recent review (Gmiter et al., 2018).

Another study showed that the idsABCDEF genes were co-expressed as an operon (Gibbs et al., 2011). It has also been shown that the IdsD protein is transported between *P. mirabilis* cells and after its transport into the cell, an interaction occurred between the IdsE protein synthesized by the cell with the obtained IdsD protein. The effects induced by the IdsD protein obtained from an unrelated cell are non-lethal (Cardarelli et al., 2015; Saak and Gibbs, 2016; Tipping and Gibbs, 2019). The role of TVISS in the Dienes phenomenon was demonstrated in 2013, within two papers, published in parallel by Wenren et al. and Alteri et al. (2013). The first study presented the idrABCDE gene cluster (idr, identity recognition), which, like the ids operon, was involved in identifying strains. Additionally, it was shown that proteins encoded by the ids and idr genes were secreted independently of each other using the TVISS apparatus (Wenren et al., 2013). The second study showed the lethal effect of proteins encoded by the pef operon (pef, hcp-vgrG1 primary effector) and secreted by TVISS on *P. mirabilis* cells. The proteins encoded by the pefD and pefE genes were responsible for identifying the relationship between strains. This study also identified a set of additional gene clusters, the products of which might potentially be transported between cells by TVISS due to the presence of the hcp and vgrG genes encoding proteins containing domains related to TVISS function. This suggests that these proteins may play a role in the formation of the Dienes line. Interestingly, it has been reported that the distribution of these gene clusters is heterogeneous between strains (Alteri et al., 2013), but no detailed analyses in this regard are available.

It is unclear whether kin discrimination plays a role in *P. mirabilis* pathogenicity during CAUTIs development. It is worth noting that TVISS may play a role as a virulence factor in various bacteria (Gallique et al., 2017), and may potentially also be involved in *P. mirabilis* virulence, since deletion of the gene encoding the TssA protein affects cell survival (as shown by studies in an in vivo mouse model), but this observation requires further research (Debnath et al., 2018).

As with other bacteria, numerous interspecies interactions are observed for *P. mirabilis*. *P. mirabilis* frequently forms a multibacterial biofilm in CAUTIs patients (Armbruster and Mobley, 2012; Moryl et al., 2013; Armbruster et al., 2018). The results of studies carried out on model systems suggest that interactions between bacteria can range from cooperation to competition, and might have serious consequences for the host. Using a mouse model of ascending UTIs, it was shown that the coexistence of *P. mirabilis* and Providencia stuartii leads to an increased incidence of urolithiasis and bacteraemia compared with monoculture. This most likely results from the synergistic induction of ureolytic activity (Armbruster et al., 2014). These observations may explain the high incidence of bacteraemia due to multimicrobial CAUTIs. It was also demonstrated that the pathogenic potential of *P. mirabilis* might be enhanced by uropathogens during polymicrobial UTIs (Armbruster et al., 2017). Although, it has been shown that the biomineralization resulting from ureolytic activity allowed for the dominance of *P. mirabilis* in a mixed biofilm with Pseudomonas aeruginosa. Such a dynamic between strains might affect the population of the biofilm formed during the development of CAUTIs (Li et al., 2021). Another example of cooperation that contributes to biofilm formation during CAUTIs is the interaction between *P. mirabilis* and Enterococcus faecalis, as both organisms were shown to successfully co-colonize the surface of catheters in a murine model of CAUTIs (Gaston et al., 2020). This work revealed that *P. mirabilis* cells can adhere to previously attached E. faecalis, which results in the formation of a robust biofilm and demonstrates the strong antibacterial resistance of coexisting strains. In contrast, Juarez et al. (2020) showed that volatile compounds produced by *P. mirabilis* (including ammonia) negatively affected the growth and biofilm formation of Klebsiella pneumoniae. Moreover, recent work showed that interspecies interactions between *P. mirabilis* and other Enterobacteriaceae may involve TVISS-independent, as yet unknown contact-dependent mechanisms (Kiani et al., 2021). Importantly, the interactions between *P. mirabilis* and other bacteria may also result in the acquisition of novel antibiotic resistance mechanisms through horizontal gene transfer (Bonnin et al., 2020).

As described above, swarming motility plays a significant role in intraspecies interactions between *P. mirabilis* strains; however, little is known about the interspecies interactions of *P. mirabilis* that involve swarming motility. Luo et al. (2018) revealed that *P. mirabilis* swarming was inhibited by haemolytic E. coli ATCC25922, and was not affected by K. pneumoniae, Acinetobacter baumannii, or P. aeruginosa strains. However, the mechanisms involved remain to be determined.

## Concluding remarks

Despite advances in our understanding of the pathogenicity of *P. mirabilis*, many aspects remain to be fully elucidated. This review focuses on the characteristics of the surface-attached microbial communities formed by *P. mirabilis* cells, and does not provide comprehensive information on other important virulence factors in this organism. There is still much to be understood regarding the process of *P. mirabilis* cell adherence, such as the role and importance of all fimbriae identified in the genome of strain HI4320. Regarding biofilm formation, the gene expression profile during biofilm maturation has not yet been investigated. We also do not know the detailed composition of *P. mirabilis* EPS or whether differences exist in the EPS produced by different strains/serogroups. Nonetheless, the mechanisms responsible for the observed natural diversity of *P. mirabilis* strains in terms of swarming ability are intriguing and appear to be vitally important in the survival and pathogenesis of the organism. Moreover, most of the mechanism mentioned within this review were investigated under laboratory conditions and its true importance in CAUTIs development still require exploration.

Both biofilm formation and swarming motility allow for intra- and interspecies interactions. The mechanisms involved in bacterial interactions and their implications for *P. mirabilis* virulence, and possibly also the biodiversity of the species, require further exploration. This concept fits with our current understanding that bacteria do not constitute single organisms, but form complex communities in which numerous interactions play an important role, affecting the biodiversity and pathogenic potential of the bacteria.

Progress in understanding *P. mirabilis* pathogenicity has resulted from the utilization of a variety of methods, including standard microbiological techniques, genetic manipulation, and microscopic imaging. However, recently, the use of next generation sequencing, at both the DNA and RNA levels, has provided deeper insight into the regulation of various processes (Armbruster et al., 2018). A comprehensive and holistic approach, including standard and new methods together with the appropriate in vitro and in vivo models of infection, will allow the important questions that remain to be addressed in the future.

## Author contributions

DG and WK contributed to conception and design of the manuscript and literature review. DG wrote the first draft of the manuscript. WK supervised the manuscript preparation. All authors contributed to manuscript revision, read, and approved the submitted version.

## Funding

Presented work was supported by grant 607 - SMGR RN. 20.115 from UJK for WK and Polish National Science Centre grant 2019/33/N/NZ6/02406 for DG.

## Conflict of interest

The authors declare that the research was conducted in the absence of any commercial or financial relationships that could be construed as a potential conflict of interest.

## Publisher’s note

All claims expressed in this article are solely those of the authors and do not necessarily represent those of their affiliated organizations, or those of the publisher, the editors and the reviewers. Any product that may be evaluated in this article, or claim that may be made by its manufacturer, is not guaranteed or endorsed by the publisher.
